# Post-treatment with maropitant reduces oxidative stress, endoplasmic reticulum stress and neuroinflammation on peripheral nerve injury in rats

**DOI:** 10.1371/journal.pone.0287390

**Published:** 2024-03-20

**Authors:** Raquel Vieira Niella, Janaína Maria Xavier Corrêa, João Felipe Ribeiro dos Santos, Larissa Ferreira Lima, Claire Souza da Costa Marques, Luciano Cardoso Santos, Larissa Rodrigues Santana, Álvaro José Chávez Silva, Keilane Silva Farias, Carlos Priminho Pirovani, Juneo Freitas Silva, Mário Sérgio Lima de Lavor

**Affiliations:** 1 Department of Agricultural and Environmental Sciences, State University of Santa Cruz, Ilhéus, Brazil; 2 Department of Biological Sciences, State University of Santa Cruz, Ilhéus, Brazil; King Saud University, SAUDI ARABIA

## Abstract

**Objective:**

To determine the effective dose and therapeutic potential of maropitant using through expression of mediators of oxidative stress, inflammatory and of the unfolded protein response (UPR) (bio) markers on spinal cord using a model of neuropathic pain induced through chronic constriction injury (CCI) in rats.

**Study design:**

Randomized, blinded, prospective experimental study.

**Animals:**

98 male Wistar rats.

**Methods:**

Rats were anesthetized with sevoflurane and after CCI, they were randomly assigned to the following groups that received: vehicle, 3, 6, 15, 30 e 50 mg/kg/24q of maropitant. The effect on inflammatory mediators (IL_10,_ TNFα), oxidative stress (GPx, CAT, SOD), microglial (IBA-1) and neuronal (NeuN, TACR1) markers was evaluated though immunohistochemistry and expression levels of markers of hypoxia (*HIF1α*, *Nrf2*), antioxidant enzymes (*Catalse*, *Sod1* and *GPx1*), and endoplasmic reticulum stress mediators (*GRP78*, *CHOP* and *PERK*) through qRT-PCR.

**Results:**

Intraperitoneal injection (IP) of maropitant inhibited nociception with ID_50_ values of 4,1 mg/kg (5,85–19,36) in a neuropathic pain model through CCI. A dose of 30 mg/kg/24q was significantly effective in reducing mechanical allodynia 1 to 4h after treatment with nociception inhibition (145,83%). A reduction in the expression of hypoxia factors (*HIF1α*, *Nrf2*) was observed, along with an increase in antioxidant activity (CAT, SOD and GPX). Additionally, there was a reduction in inflammatory markes (IL_10,_ TNFα), microglial (IBA-1), and neuronal markers (NeuN, TACR1).

**Conclusion and clinical relevance:**

These findings demonstrate that the determined dose, administered daily for seven days, had an antinociceptive effect, as well as anti-inflammatory and antioxidant activity.

## Introduction

Neuropathic pain is a severe and chronic condition that may develop due to damage to the peripheral and/or central somatosensory system, resulting in abnormal nociceptive responses [1, 2].

Epidemiologic surveys have revealed that neuropathic pain may persist in 8% of the overall population, leading to financial losses for both individuals and society [3, 4]. Unfortunately, currently available drugs, including anticonvulsants, antidepressants and opioids, are insufficiently effective in treating pain and have been found to exhibit side effects. Therefore, new therapeutic strategies are needed to effectively contribute to multimodal treatment for this complex, yet not completely understood, condition that impacts the quality of life for both humans and non-human animals. Neuropathic pain can contribute to or lead to anxiety, depression, loss of memory and neurodegenerative diseases [5–7].

Various physiological and pathophysiological processes in mammals are modulated by the interaction of substance P (SP), the most important member of the tachykinin family, with the neurokinin 1 receptor (NK-1R). NK-1R antagonists promote, in a dose-dependent manner, a range of effects such as anticonvulsant, antipruritic, anti-inflammatory, antitumor, antimetastatic, antidepressant, analgesic, anti-emetic, anti-angiogenic, apoptotic, antiviral or anxiolytic effects [6].

Maropitant, a selective antagonist to the NK1 receptor, is widely used as an antiemetic in dogs [7, 8] and cats [9, 10]. Research has demonstrated its promising use in the treatment of acute pain in these animals [11–13], and there is potential to explore new treatment applications across other models and experimental species.

In relations to the activity of the substance P (SP) and NK1 receptor involved in the process of pain transmission, studies have shown the safety and pharmacokinetics of intraperitoneal injection of maropitant in rats, including its anti-inflammatory capacity [14, 15].

In this context, inhibiting the action of the SP using an NK1 antagonist is expected to be beneficial in modulating the neuroinflammatory process of chronic pain, justifying its research as a potential treatment for neuropathic pain [16]. As maropitant is an NK1 receptor antagonist, it is anticipated to provide an analgesic effect by blocking the pharmacological action of the SP in the spinal cord and the brain.

Therefore, this study aimed to determine the effective dose of maropitant on the mechanical nociceptive threshold (MNT) and to evaluate its therapeutic potential in the spinal cord using a model of neuropathic pain induced by chronic constriction injury in rats. The findings demonstrated that daily maropitant treatment post-neuropathy was capable of increasing MNT, the expression of antioxidant enzymes in spinal cord, and inducing a positive modulation of oxidative stress, endoplasmic reticulum stress and immune mediators. This characterization marks the first exploration of the therapeutic potential of the NK1 antagonist in neuropathic pain, to the best of the authors knowledge.

## Materials and methods

### Animals

98 male adult Wistar rats (*Rattus norvegicus*) weighing 200 – 250g and aged two-month, were included in this study. The animals were supplied by Santa Cruz State University and housed in plastic cages in a controlled environment (12:12 light/dark cycle; 22 ± 2°C; humidity 50 ± 5%). They received water daily and fed *ad libitum* with commercial rodent food. All rats underwent a two-week acclimatization period. This study was conducted in strict accordance with the recommendations outlined in the Guide for the Care and Use of Laboratory Animals of the National Institute of Health and the use of the smallest number of animals per group necessary to obtain statistically reliable data aligns with ethical principles. It received approval from the Ethics Committee on the Use of Animals (ECUA) at the State University of Santa Cruz under protocol number 027/2015. Throughout the experiment, trained staff monitored the animals for potential signs of pain and discomfort, ready to intervene if necessary.

### Measurement of mechanical withdrawal threshold

After the pre-experimental adaptation period, the animals underwent baseline assessment of the mechanical nociceptive threshold (MNT) using a digital analgesiometer (EFF-301, Insight Equipment). The assessment involved the method of increasing pressure, largely described by Cunha et al. [17]. The pressure recorded on the device at the moment of the animal’s reflex was considered the MNT. Mechanical sensitivity was calculated as the average of at least three measurements, conducted at 5-minute intervals for each measurement. All measurements were performed by, the same evaluator, who had undergone prior training.

### Chronic constriction injury of sciatic nerve (CCI)

Peripheral neuropathic pain was induced using a chronic constriction injury (CCI) model of the sciatic nerve as described by Bennet and Xie [18]. The animals underwent inhalational general anesthesia with sevoflurane (Cristalia Prod. Quım. Farm. Ltda, Brazil) to perform the CCI.

An incision was made in the gluteal region of the skin on the right posterior limb, followed by dissection of the femoral biceps muscle. The sciatic nerve was exposed proximal to its trifurcation and sutures were applied around using polyamide suture (5–0), while the musculature and skin were sutured with polyamide suture (4–0). As a prophylactic antibiotic therapy, all animals received cephalothin sodium (Ceflen®, Agila Especialidades Farmacêuticas Ltda, Brazil) at a dose of 60mg/kg/SID, subcutaneously.

The development of neuropathy was evidenced after seven days, assessed using a digital analgesiometer to measure the reduction in the MNT on the first day of neuropathy, marking the commencement of treatments.

### Experimental design

**Fig 1:**
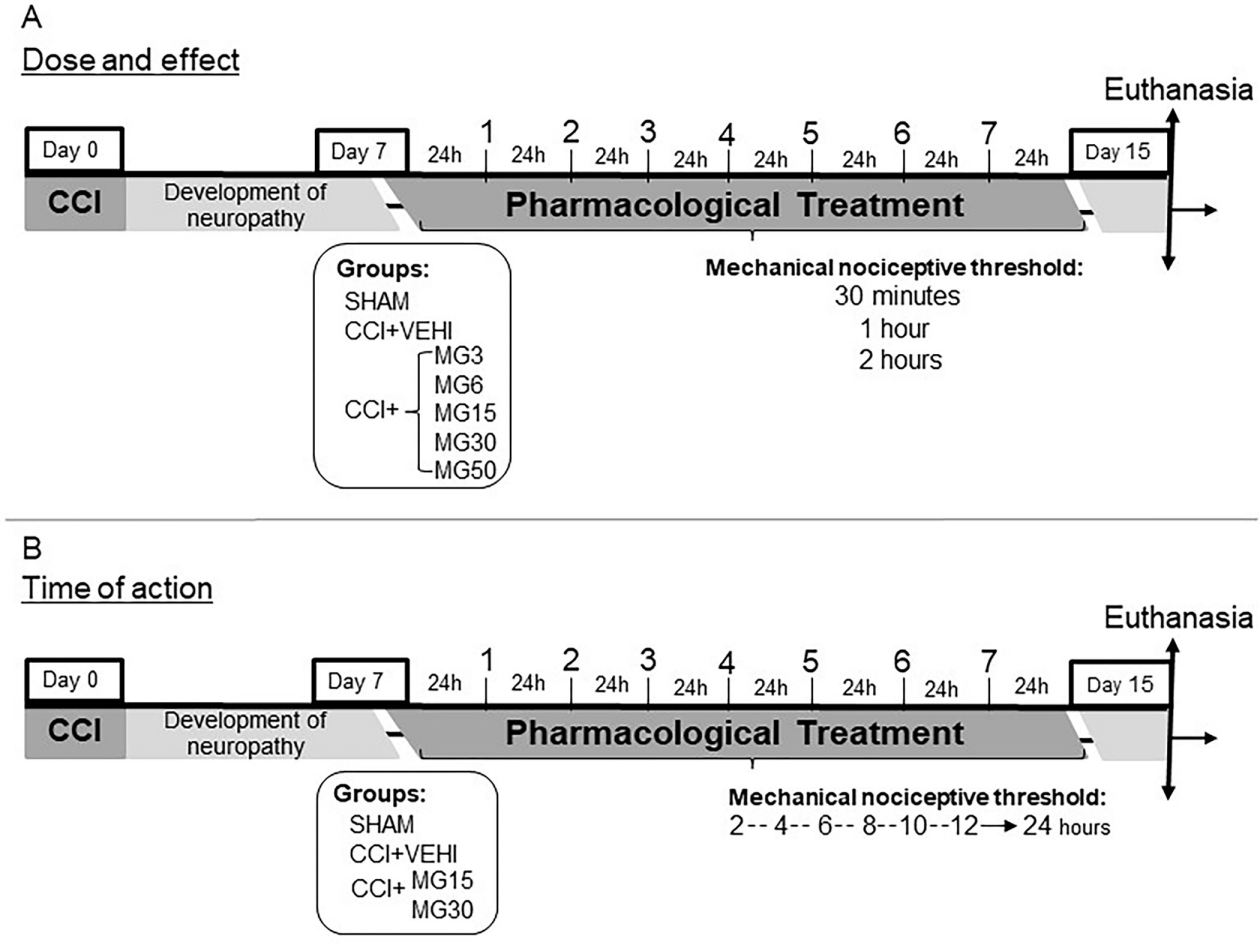
Experimental treatment protocol and evaluations to define dose and time of action.

Seven groups, each comprising seven Wistar rats, were included in the present study. Animals were treated with maropitant citrate (Cerenia®, Pfizer Animal Health, NY, USA) every 24 hours, injected intraperitoneally (IP) for 7 consecutive days with (Fig 1A).

SHAM = nerve exposure and 3 ml/kg/24q (0.9% NaCl);

CCI+VEHI = CCI+3ml/kg/24q NaCl 0,9%;

CCI+MG3 = CCI+maropitant 3mg/kg/24q;

CCI+MG6 = CCI+maropitant 6mg/kg/24q;

CCI+MG15 = CCI+maropitant 15mg/kg/24q;

CCI+MG30 = CCI+maropitant 30mg/kg/24q;

CCI+MG50 = CCI+maropitant 50mg/kg/24q.

The evaluation of the MNT was carried out at 30 minutes, one hour and two hours after application of the treatment. After analyzing the preliminary results and defining the dose with the best clinical efficacy, a monitoring curve of the duration of action of maropitant was performed in the animals of the SHAM, CCI+VEHI, MG15, and MG30 groups, during 24 hours after treatment application, at two-hour intervals (Fig 1B).

**Fig 2:**
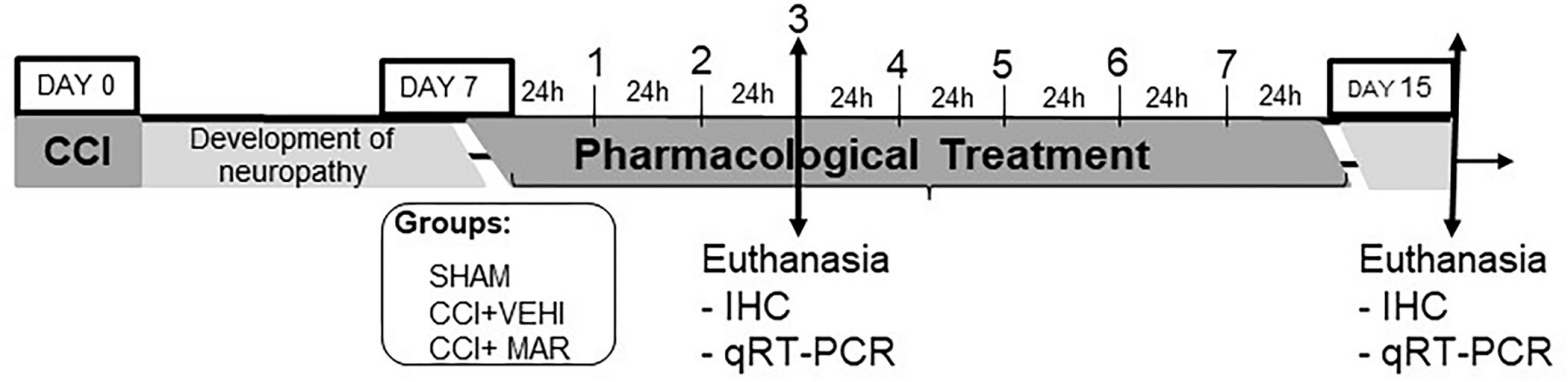
Experimental treatment protocol with defined dose.

Also, the effect of treatment with maropitant 30mg/kg/24q on the spinal cord was verified. Animals were randomly assigned to blocks divided into 6 groups (n = 7). After 72 hours, and 7 days of treatment, rats were euthanized with propofol overdose (Propotil® 1%, Bio Chimico®) and spinal cord tissues from the lumbar segment were collected for further analysis (Fig 2).

The experiment took place over 29 days, including adaptation of the animals, measurement of baseline MNT, surgical procedure, treatment and evaluations, and euthanasia.

### Tissue processing and histopathological evaluation

Lumbosacral spinal cord segments were collected using the hydropropulsion method, immediately fixed in 10% formaldehyde for 24 hours and processed using the paraffin inclusion method. Tissue sections of 4 μm thickness were then prepared on gelatinized histological slides for immunohistochemistry.

### Immunohistochemistry (IHC)

Histological sections of the spinal cord were prepared for immunohistochemical analysis using anti-CAT (sc-271803), anti-SOD (sc-101523), anti-GPx (sc-133160), anti-TNFα (sc-527), anti- IL_10_ (sc-365858), anti-TACR1 (sc-365091), anti-NeuN (sc-166824) and anti-IBA-1 (sc-32725) from Santa Cruz Biotechnology, CA, USA. All antibodies used in this study were validated by the manufacturer.

The streptavidin-biotin-peroxidase technique (Streptavidin Peroxidase, Lab Vision Corp., Fremont, CA, USA) was employed with antigenic recovery in a water bath at 98°C in citric acid (P.M. 192.13 pH 6.0). Subsequently, samples underwent a 30 minute blocking of endogenous peroxidase and serum (Ultra vision Block, Lab Vision Corp.®, Fremont, CA. USA), followed by an overnight incubation with the primary antibody. The secundary antibody incubation lasted for 45 minutes, and blocking with streptavidin peroxidase for an additional 30 minutes. Diaminobenzidine (DAB Substrate system, Lab Vision Corp., Fremont, CA. USA) served as the chromogen. Finally, the sections were counterstained with Harris hematoxylin. A negative control was established by substituting the primary antibody with PBS [19].

For quantitative analysis, the immunostaining area was determined using WCIF ImageJ® software (Media Cybernetics Manufacturing, Rockville, MD, USA). Random images were captured from five regions of the spinal cord under a Leica DM 2500 microscope using a Leica DFC 295 digital camera (Leica Microsytems, Germany). Color deconvolution and thresholding of the images were performed for the analysis. The data of each tissue were then archived, analyzed, and expressed as the immunostaining area in pixels [19].

### Real-time PCR qRT-PCR

For the qRT-PCR technique, total RNA from the spinal cord was extracted using TRIzol® according to the manufacturer’s instructions (Invitrogen, Life Technologies, Carlsbad, CA, USA). Subsequently, 1 μg of RNA was used for the reverse transcription reactions with the GoTaq® qPCR and RT-qPCR Systems kit (A6010, PROMEGA). The transcripts of the target genes were quantified by qPCR using SYBR Green in the Applied Biosystems® 7500 Real-Time PCR System. For the reactions, 1.5 μL of cDNA, 100 nM of each primer, and 10 μL of the GoTaq® qPCR Master Mix 2X reagent was used in a final volume of 20 μL of reaction. As a negative control, the DNA amplification mix was used, in which the cDNA sample was replaced by ultrapure water. The amplifications were performed under the following conditions: enzyme activation at 95°C for 2 min, 40 cycles of denaturation at 95°C for 15 s, and annealing/extension at 60°C for 60 s. To evaluate the linearity and efficiency of qPCR amplification, standard curves of all transcripts were generated using serial dilutions of the cDNA, followed by an evaluation of the melting curve of the amplification products. The primers for *Sod1*, *Catalase*, *Gpx1*, *Hif1a*, *Grp78*, *Chop*, *Perk* and *Nrf2* were delineated based on *Rattus norvegicus* mRNA sequence (Table 1). Gene expression was calculated by the 2-DDCT method, where the results obtained for each group were quantitatively compared after normalization based on the expression of GAPDH *Rattus norvegicus*.

**Table 1:** List of genes and nucleotide sequences for qPCR primers.

Gene	Sequence (5’-> 3’)	Accession number
Sod 1	Forward: GAAAGGACGGTGTGGCCAAT Reverse: CTCGTGGACCACCATAGTACG	NM_017050.1
Catalase	Forward: CTGACTGACGCGATTGCCTAR Reverse: GTGGTCAGGACATCGGGTTT	NM_012520.2 R
Gpx1	Forward: GCGCTACAGCGGATTTTTGA Reverse: GAAGGCATACACGGTGGACT	NM_030826.4
Hifα	Forward: AGCAATTCTCCAAGCCCTCC Reverse: TTCATCAGTGGTGGCAGTTG	NM_024359.1
Grp78	Forward: TGAAGGGGAGCGTCTGATTG Reverse: TCATTCCAAGTGCGTCCGAT	NM_013083.2
Chop	Forward: TGGCACAGCTTGCTGAAGAG Reverse: TCAGGCGCTCGATTTCCT	NM_001109986.1
Perk	Forward: GGCTGGTGAGGGATGGTAAA R: TTGGCTGTGTAACTTGTGTCATC	NM_031599.2
Nrf2	Forward: CCCATTGAGGGCTGTGATCT Reverse: GCCTTCAGTGTGCTTCTGGTT	NM_031789.2
Gapdh	Forward: GCGCTACAGCGGATTTTTGA Reverse: GAAGGCATACACGGTGGACT	NM_031797.2

### Statistical analysis

Statistical analysis were conducted using the Graph Pad Prism Software version 5.01. A completely randomized design was employed, and the results were expressed as the mean ± standard error of the mean. Data of the mechanical nociceptive threshold were analyzed through the analysis of variance method (ANOVA) followed by the Bonferroni test, and for comparison between two groups, Student’s t test was used or Student-Newman-Keuls test (SNK) for multiple comparisons of immunostaining data. For all analysis, the differences were considered significant when p < 0.05.

## Results

### The effect of maropitant treatment on mechanical withdrawal threshold, effective dose determination and time of action

The CCI resulted in significant development of mechanical hyperalgesia, demonstrated through reduction of MNT in the animals seven days after surgical procedure.

**Fig 3:**
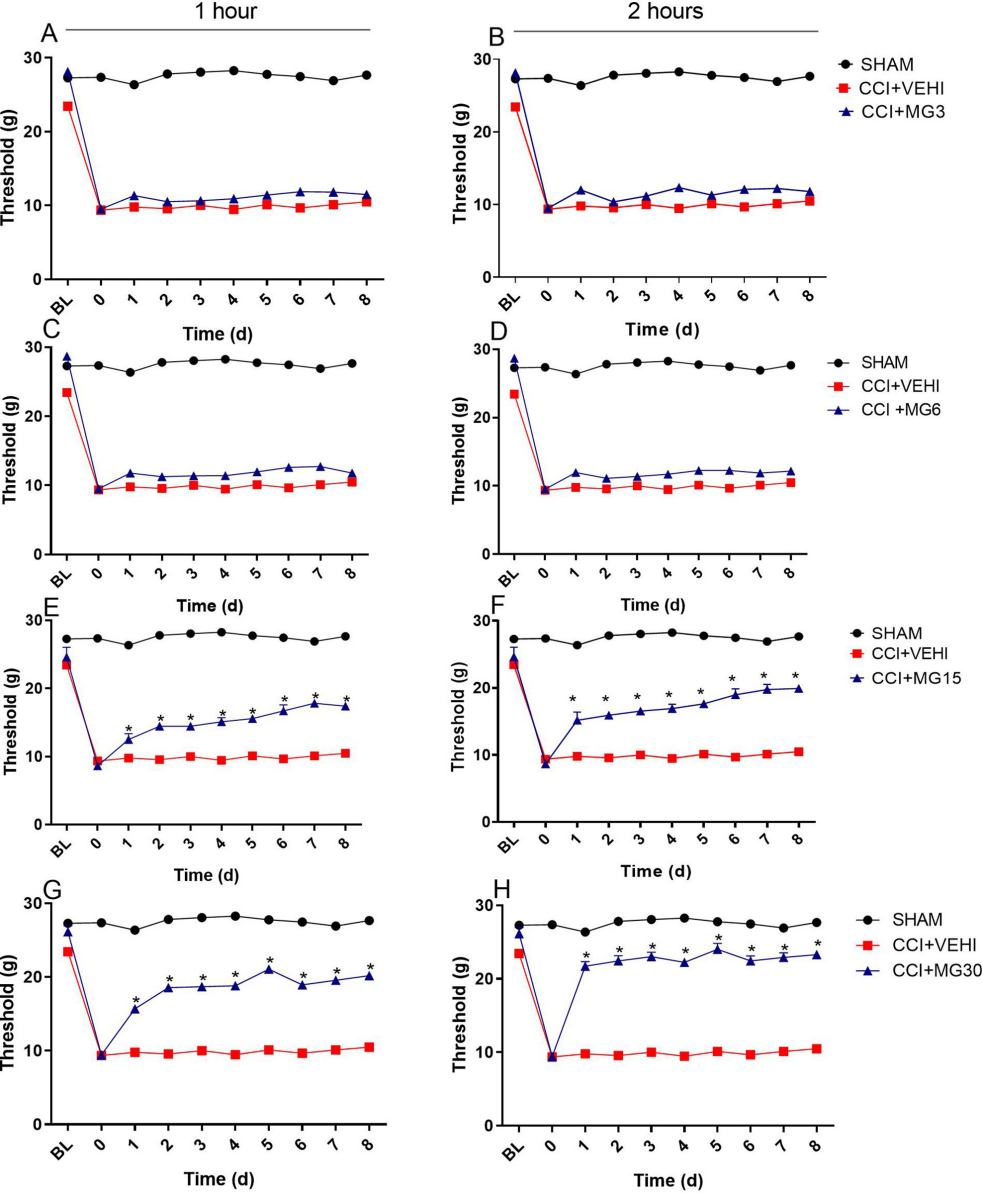
Evaluation of the mechanical nociceptive threshold with a digital analgesiometer for eight days, after one and two hours of treatment application, in different doses. BL = basal; 0 = neuropathy. Values are expressed as mean ± SEM. *p<0,05 compared to CCI+VEHI.

Doses of 3 and 6mg/kg/24q showed no significant difference (p>0.05) compared to CCI + VEHI during the three evaluation moments throughout the experimental period (Fig 3A–3D).

After one hour of injection, maropitant 15 and 30mg/kg/24q groups showed significant increase on MNT when compared to CCI + VEHI (p<0.001). Although, the doses of 15 and 30 mg/kg/24q were not different at this time of assessment (p>0.05) (Fig 3E and 3G), both were able to increase MNT with antinociceptive effect of 11.2±0.54% and 13.9%±0.46%, respectively. However, after two hours of application, they differed; 30mg/kg/24q provided a higher mechanical nociceptive threshold than 15mg/kg/24q (p<0.001).

**Fig 4:**
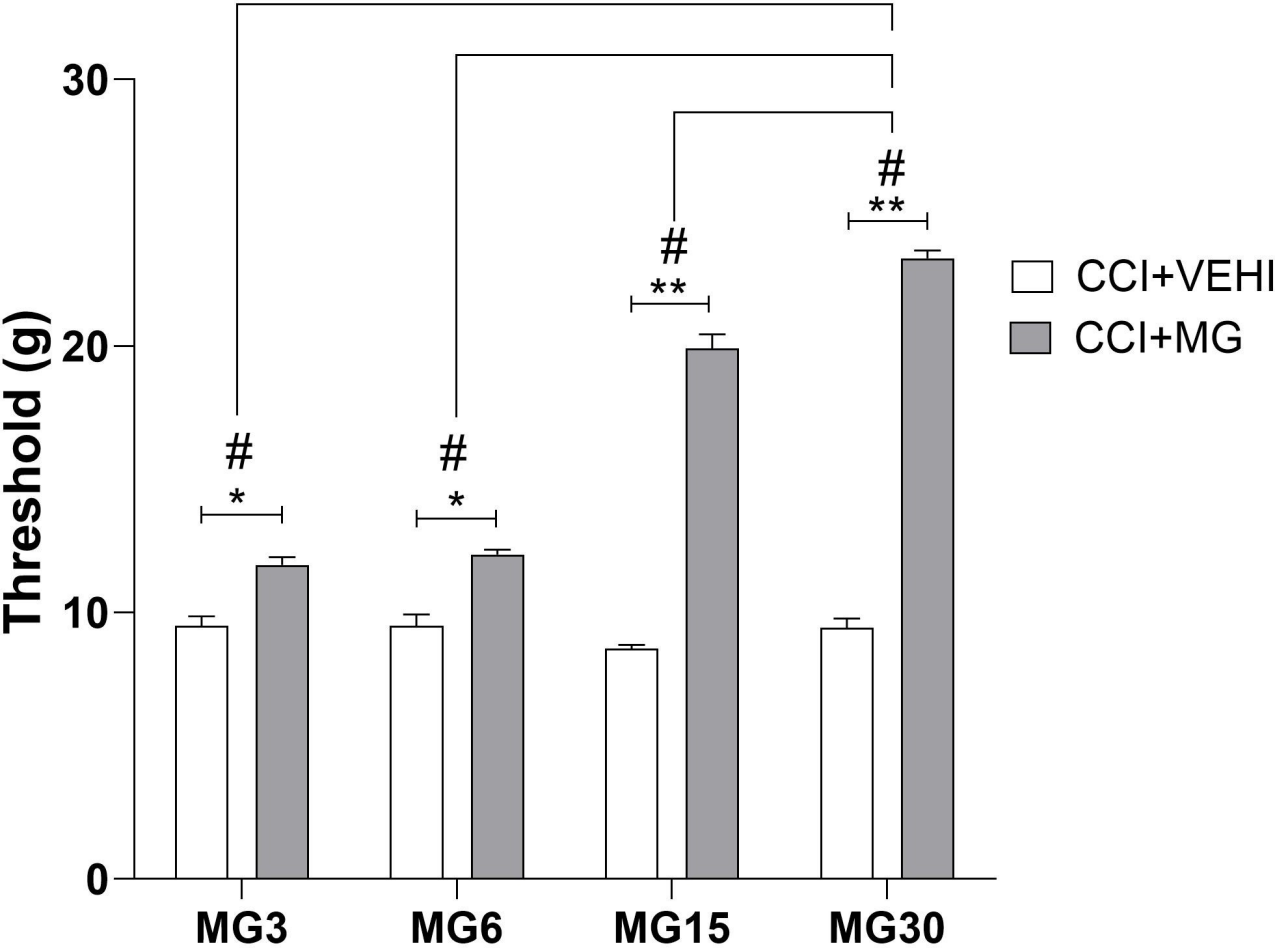
Efficacy of maropitant doses. CCI+VEHI = positive control group. CCI+MG = maropitant groups. Values are expressed in mean ± SEM. *p<0.01, **p<0.0001, compared between groups).

The MNT obtained at the end of the treatment were (mean ± SEM) 11.79±0.28; 12.17±0.18; 19.91±0.52 and 23.28±0.29 in the maropitant 3, 6, 15 and 30mg groups, respectively. The ED50 (effective dose that produces a specific effect in 50% of the population) was 4.1mg/kg (2.9–5.7). In addition, the increase observed in MNT from baseline was 23.96%; 28.28%; 130.33% and 145.83% for 3, 6, 15 and 30mg, respectively (Fig 4).

A 100% lethality rate was observed in animals treated with 50mg/kg/24q (7 animals). On average, fifteen minutes after the initial application, animals exhibited signs of prostration, respiratory depression, and generalized tremors, culminating in cardiac arrest.

Monitoring the MNT for 24 hours after application, allowed us to determine the duration of the medication’s action in this species.

**Fig 5:**
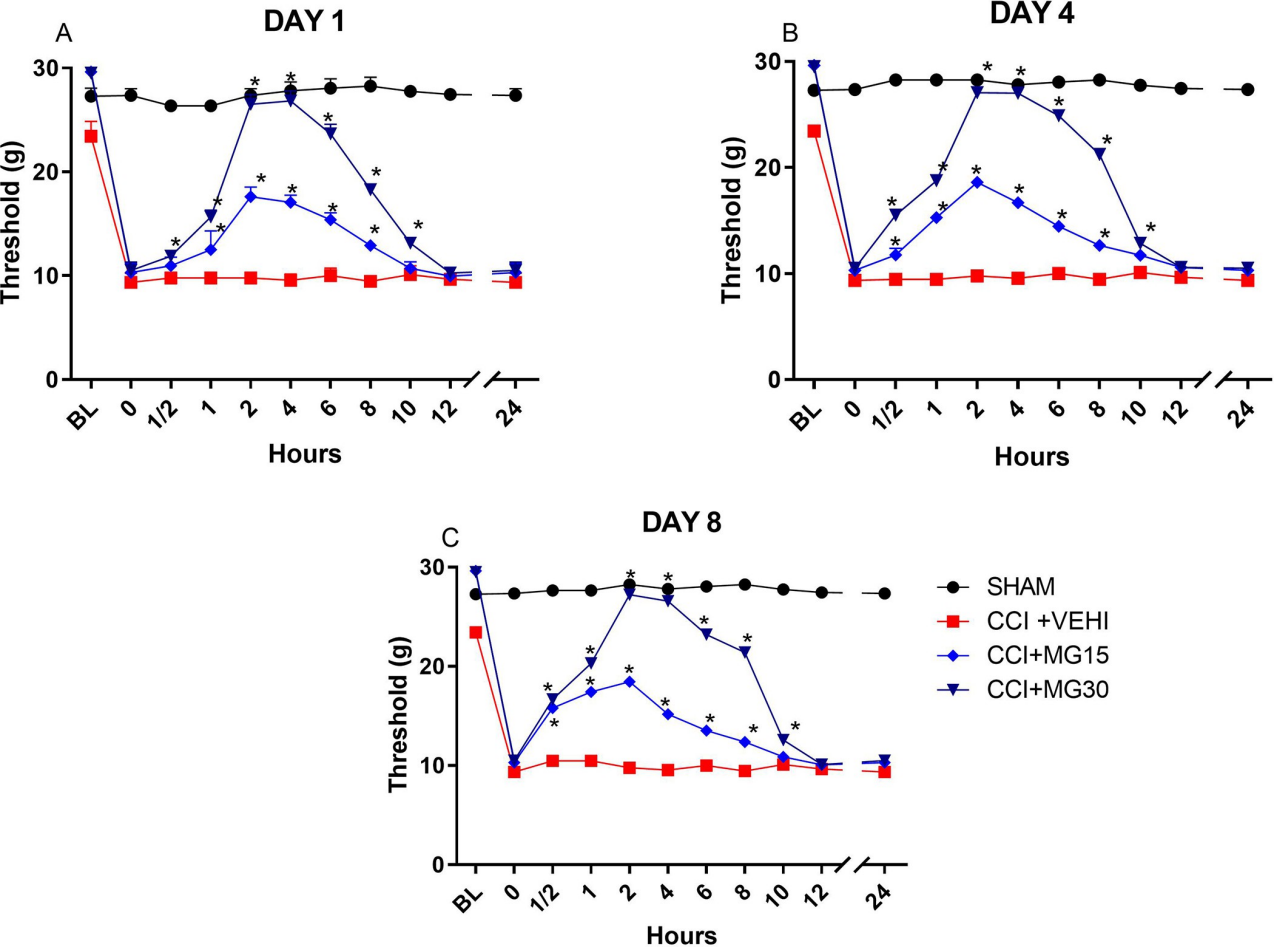
Evaluation of mechanical nociceptive threshold using a digital analgesiometer for 24 hours after treatment application. (A) Day 1 of treatment; (B) Day 4 of treatment; (C) Day 8 of treatment. BL = basal; 0 = neuropathy. Values are expressed in mean ± SEM. *p<0,05 compared to CCI+VEHI.

Animals from MG3MNT increased up to 10 hours (p<0.001), with a peak between 2–4 hours, while animals from MG15 preserved the nociceptive threshold increase for 8 hours (p< 0.001) (Fig 5).

### Maropitant treatment enhaces the medullary expression of antioxidant enzymes in rats with neuropathic pain

Given the association of oxidative stress with neuropathic pain, and the reported oxidative damage in rat spinal cord following chronic constriction injury (CCI) of the sciatic nerve, its becomes essential to assess whether daily maropitant treatment could mitigate or reverse the oxidative stress induced by this neuropathy. In pursuit of this, the gene and protein expression profile of *Hif1α* and *Nrf2*, transcriptional biomarkers involved in the expression of antioxidante enzymes under hypoxic conditions, were examined.

**Fig 6:**
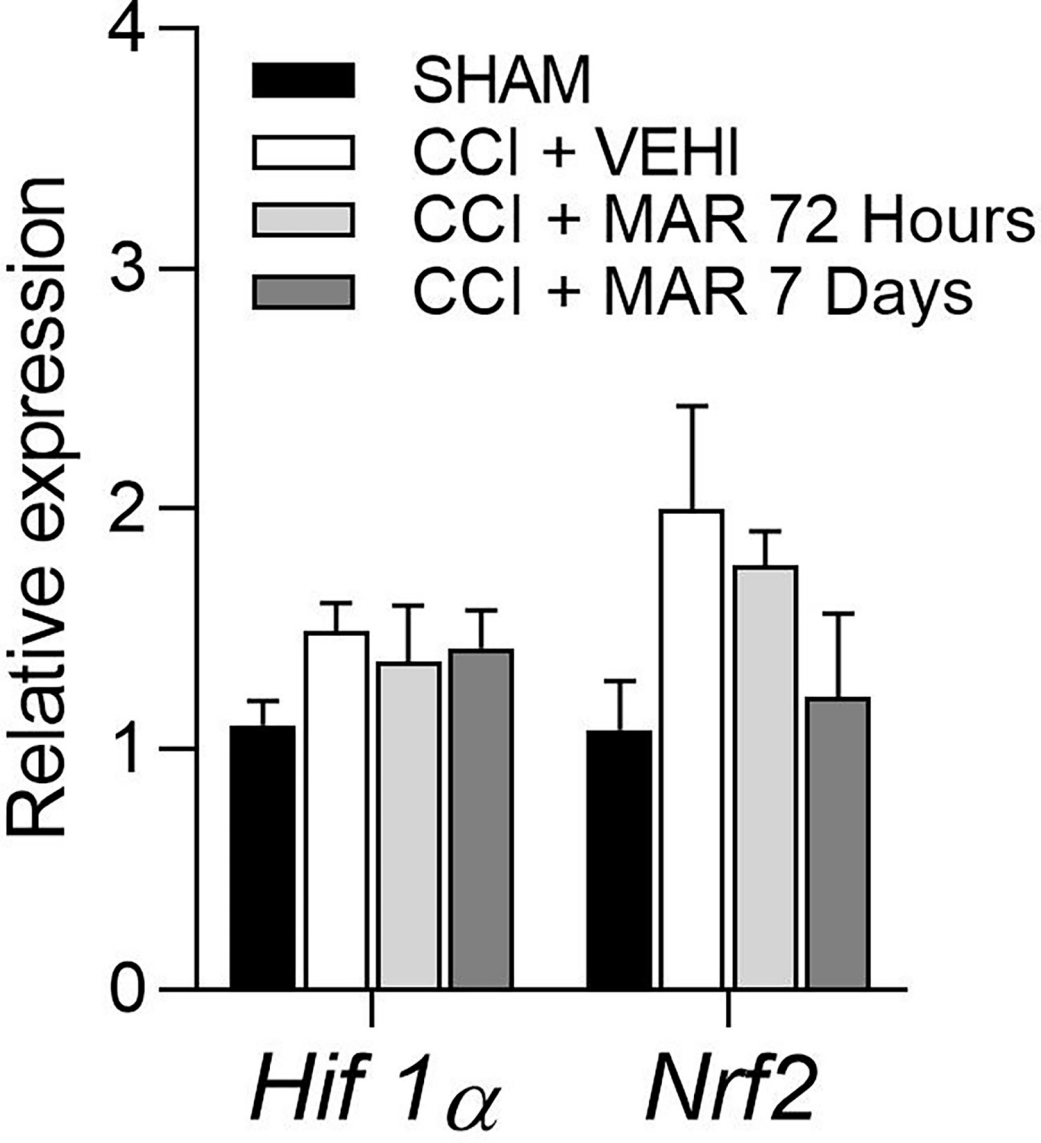
Expression of *HIF1α* and *Nrf2* in the spinal cord of sham, control positive and rats treated with maropitant after sciatic nerve injury. Relative gene expression of *Hif1α* and *Nrf2* (mean ± SEM; n = 7).

The analysis revealed that sciatic nerve ligation increased the medullary expression of *Hif1α* and *Nrf2* transcripts comparted to the negative control group (SHAM). However, the groups treated with maropitant exhibited lower mRNA expression compared to positive control group (CCI+VEHI) (Fig 6).

**Fig 7:**
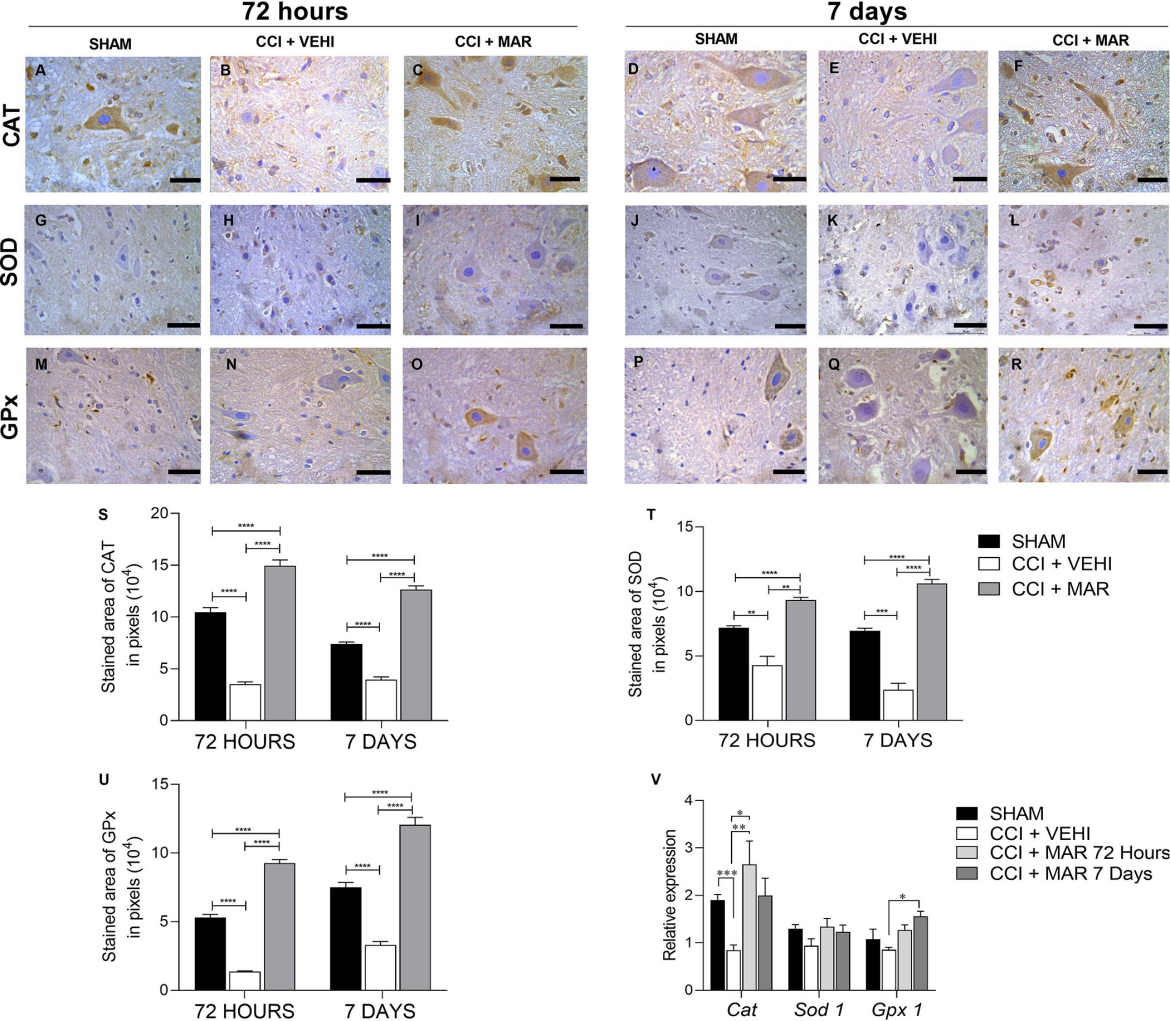
Expression of CAT, SOD and GPx in spinal cord of rats treated with maropitant after sciatic nerve injury. (A-F) Photomicrographs of immunohistochemical expression of catalase (Streptavidin-biotin-peroxidase; Harris hematoxylin; Bar = 50 μm). (G-L) Photomicrographs of the immunohistochemical expression of SOD (Streptavidin-biotin-peroxidase; Harris hematoxylin; Bar = 50 μm). (M-R) Photomicrographs of the immunohistochemical expression of GPx (Streptavidin-biotin-peroxidase; Harris hematoxylin; Bar = 50 μm) (S) Immunolabeling area, in pixels, of SOD expression (mean ± SEM; n = 7). (T) Immunolabeling area, in pixels, of SOD expression (mean ± SEM; n = 7). (U) Immunolabeling area, in pixels, of GPx expression (mean ± SEM; n = 7). (V) Relative gene expression of *Catalase*, *Sod1* and *Gpx 1* in the spinal cord (mean ± SEM; n = 7).

Regarding the expression of antioxidant mediators in the spinal cord, immunohistochemistry analysis showed that CAT expression was reduced in the injured group without treatment at 72 hours, and 7 days, showing a lesser expression of immunopositive cells (Fig 7B and 7E), with significant difference from the compared groups. The treatment with maropitant significantly increased the area of protein expression CAT (p<0.0001) (Fig 7C and 7F), with values similar to SHAM group (Fig 7A and 7D) when treated for 7 days (Fig 7S).

Immunoreactivity to SOD was present in all experimental groups (Fig 7G–7L). A significantly lower expression of SOD was observed in the animals from CCI + VEHI group, in 72 hours (Fig 7H) and seven days (Fig 7K). Treatment with maropitant was able to increase expression of SOD in both evaluated moments (Fig 7I and 7L) and proved to be more accentuated after seven days of treatment (Fig 7T).

Non-treated animals that underwent CCI showed lesser expression of GPx (Fig 7N and 7Q) when compared to SHAM group (Fig 7M and 7P) (p<0.0001). There was a significant increase in the intensity of expression of such biomarker, on animals treated with maropitant (Fig 7O and 7R) (p<0.0001) accentuated at 7 days.

Furthermore, *Catalase* and *Gpx1* gene transcription expression showed higher mRNA values in the maropitant-treated groups, compared to CCI+VEHI (p = 0,0083 and p = 0,020) while meeting SHAM mRNA expression values (Fig 7V).

### Maropitant treatment inhibits the expression of reticular stress mediators in the spinal cord of rats with neuropathic pain

Given that studies have shown that neuropathic pain induces endoplasmic reticulum stress in the spinal cord of rats, we investigated whether treatment with maropitant could reverse this process. The expression of key mediators in the unfolded protein response (UPR) activation pathway—*GRP78*, *CHOP* and *PERK*–was analyzed, indicating the occurrence of endoplasmic reticulum stress.

**Fig 8:**
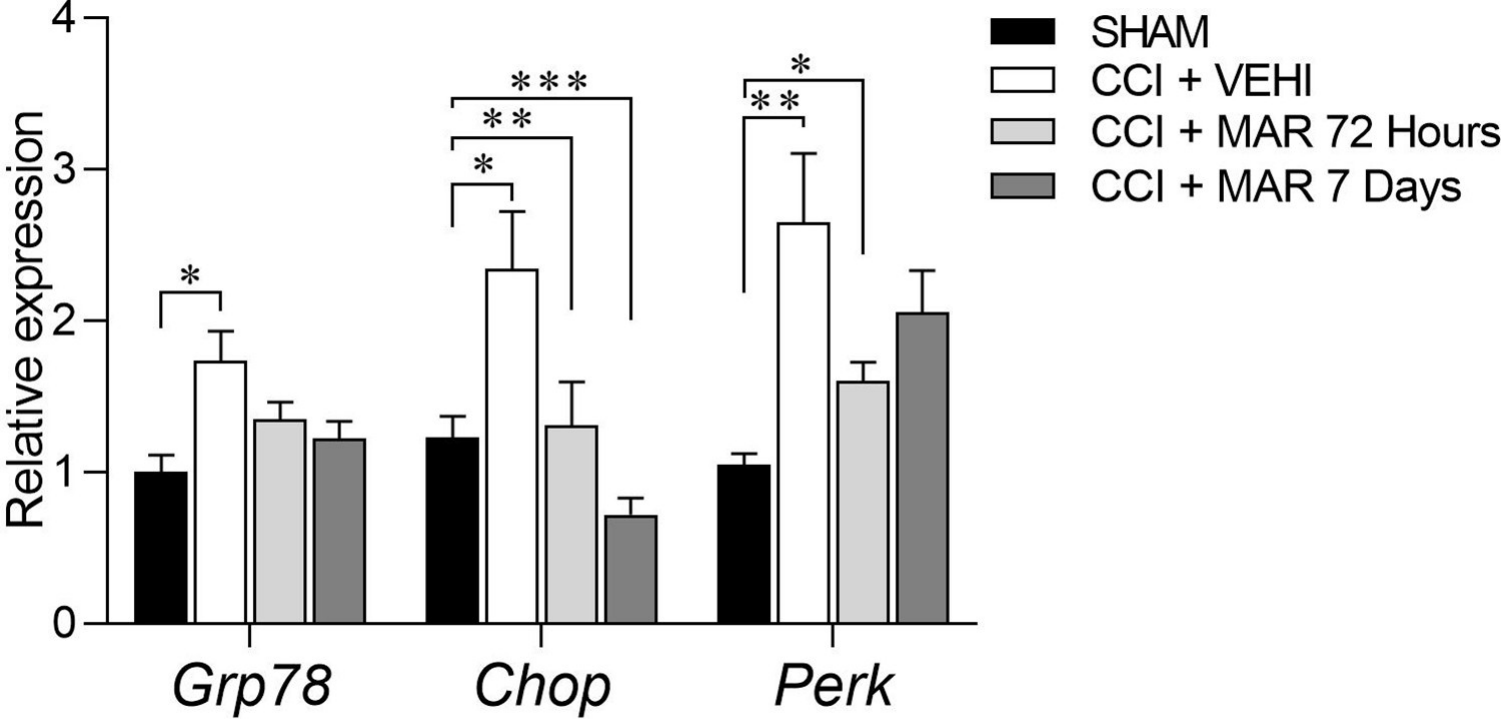
Expression of GRP78, CHOP and PERK in the spinal cord of sham, control positive and rats treated with maropitant after sciatic nerve injury. Relative gene expression of *Grp78*, *Chop* and *Perk* (mean ± SEM; n = 7).

The relative expression of *GRP78*, *CHOP* and *PERK* genes was found to be increased in CCI+VEHI compared to SHAM (p<0.05); treatment with maropitant was able to reverse augmented expression, caused by chronic constrictive injury, resembling the expression of the SHAM group (Fig 8) at the end of treatment.

### Treatment with maropitant alters the expression of spinal cord IL_10_, TNFα and microglial marker (IBA-1) in rats with neuropathic pain

Considering that the development of neuropathic pain can alter the immune system mediators, we evaluated the effect of daily treatment with maropitant on IL_10_, TNFα and its influence on medullary microglial activity expression in rats with sciatic nerve injury.

**Fig 9:**
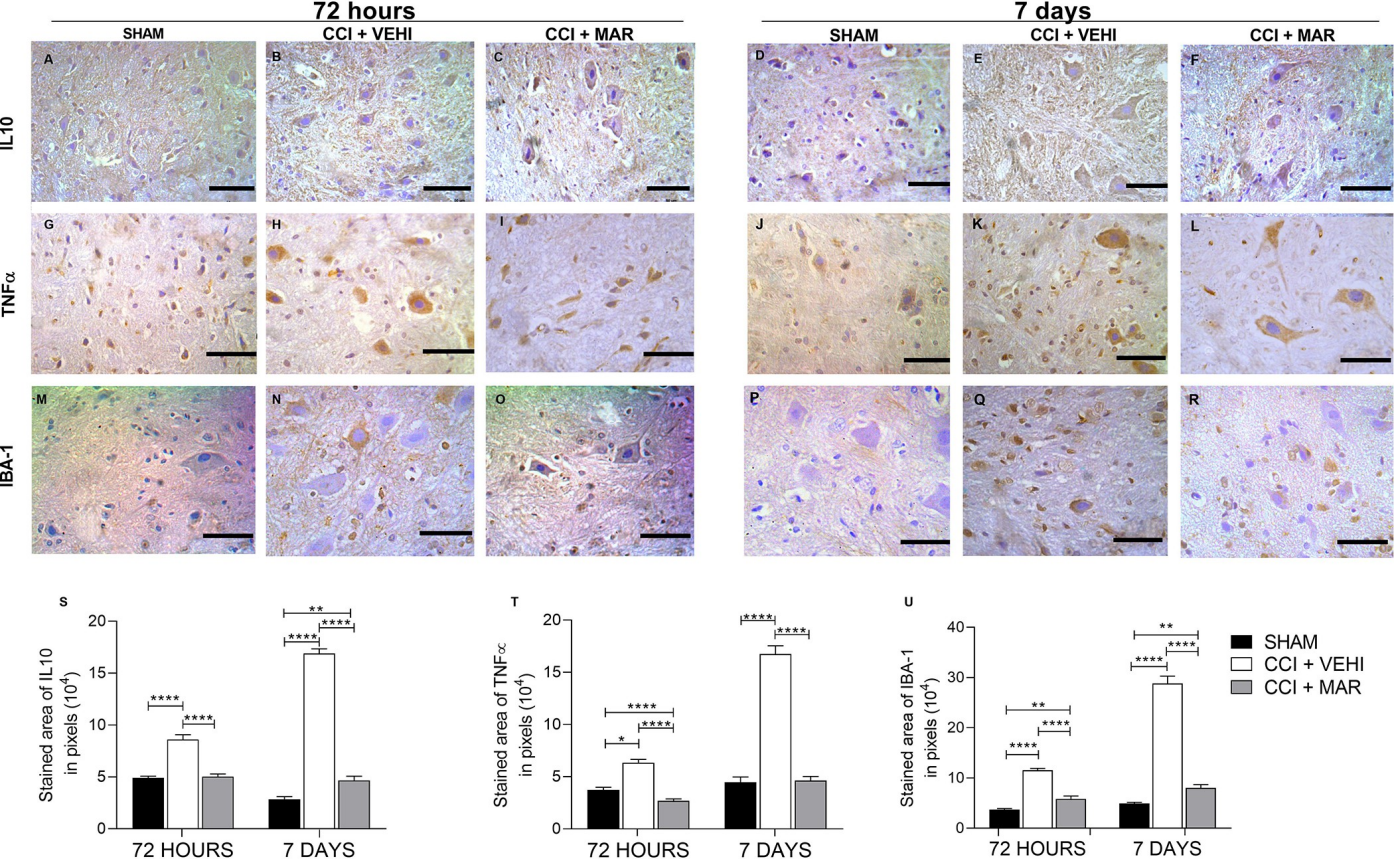
Expression of IL_10_, TNFα and IBA1 in spinal cord of rats. (A-F) Photomicrographs of immunohistochemical expression of IL_10_ (Streptavidin-biotin-peroxidase; Harris hematoxylin; Bar = 50 μm). (G-L) Photomicrographs of the immunohistochemical expression of TNFα (Streptavidin-biotin-peroxidase; Harris hematoxylin; Bar = 50 μm). (M-R) Photomicrographs of the immunohistochemical expression of IBA-1 (Streptavidin-biotin-peroxidase; Harris hematoxylin; Bar = 50 μm) (S) Immunolabeling area, in pixels, of IL_10_ expression (mean ± SEM; n = 7). (T) Immunolabeling area, in pixels, of TNFα expression (mean ± SEM; n = 7). (U) Immunolabeling area, in pixels, of IBA-1 expression (mean ± SEM; n = 7, ***p<0,001; ****<0,0001).

Regarding the inflammatory mediators in the dorsal horn of the spinal cord, IL_10_ and TNFα exhibited similar protein expression in the experimental groups (Fig 9A–9L). However, immunostaining for IL_10_ and TNFα was more intense in the CCI+VEHI group in both moments (72 hours and 7 days), compared to SHAM group (p<0,0001) (Fig 9A and 9B, 9D and 9E, 9G and 9H, 9J and 9K), as confirmed by immunolabeling area analysis (Fig 9S and 9T). Treatment with maropitant reduced immunostaining of both IL_10_ and TNFα, at 72 horas and 7 days (Fig 9C, 9F, 9I and 9L).

Immunoreactivity for IBA-1 was present in all experimental groups, showing significant increase in the expression of IBA-1 in the spinal cord of the animals from CCI + VEHI (Fig 9N and 9Q), compared to SHAM group (Fig 9M and 7P), on the evaluated times (72 hours and 7 days) (p<0.001), indicating greater microglial activation on the former. Treatment with maropitant significantly decreased the number of activated microglia (Fig 9O and 9R) when compared to the control group, treated with vehicle (Fig 9U) (p<0.001).

### Maropitant treatment affects the expression of neuronal markers and NK1r in the spinal cord of rats with neuropathic pain

**Fig 10:**
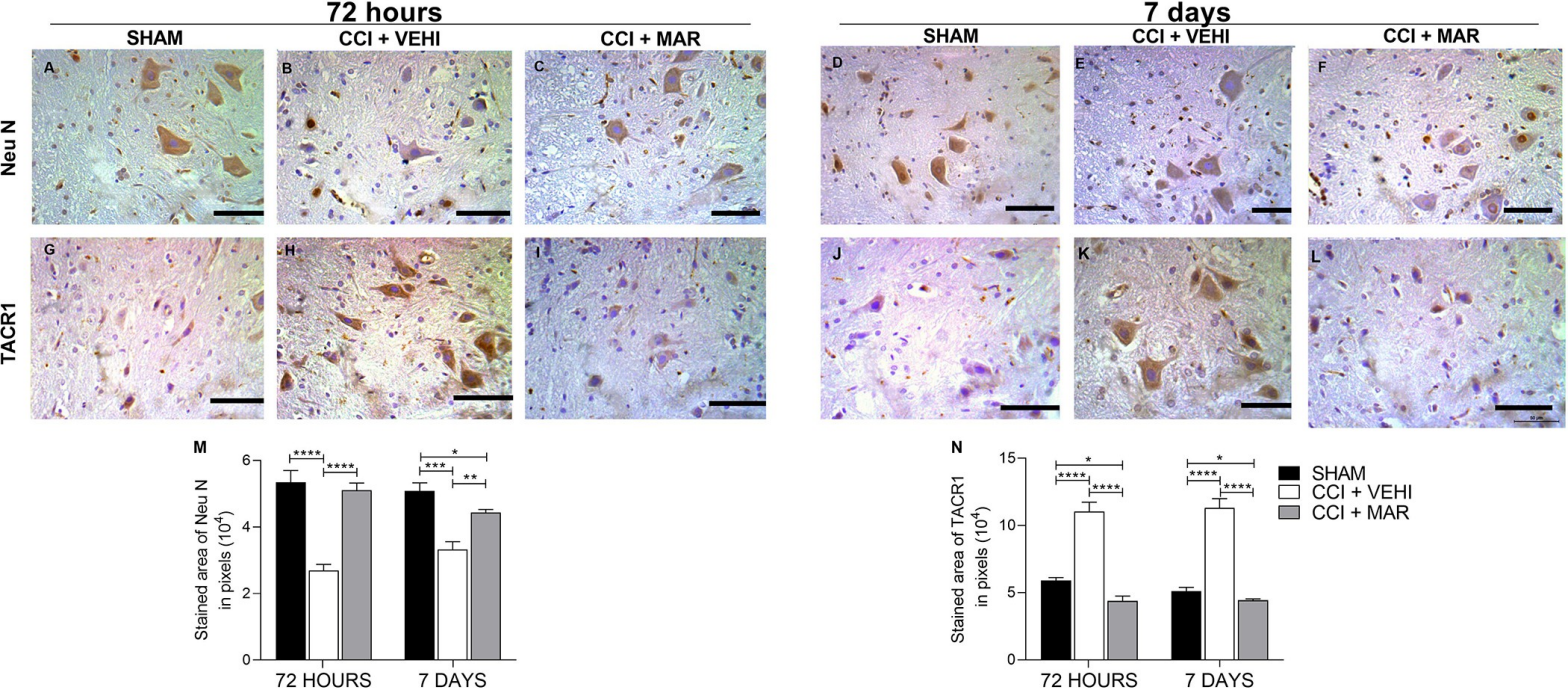
Expression of NeuN and TACR1 n the spinal cord of rats. (A-F). A-F) Photomicrographs of immunohistochemical expression of NeuN (Streptavidin-biotin-peroxidase; Harris hematoxylin; Bar = 50 μm). (G-L) Photomicrographs of the immunohistochemical expression of TACR1 (Streptavidin-biotin-peroxidase; Harris hematoxylin; Bar = 50 μm). (M) Immunolabeling area, in pixels, of NeuN expression (mean ± SEM; n = 7). (N) Immunolabeling area, in pixels, of TACR1 expression (mean ± SEM; n = 7, ***p<0,001; ****<0,0001).

Almost all neuronal cell populations exhibited immunoreactivity for NeuN (Fig 10A–10F). Staining was present in both nuclei and cytoplasm, extending to neuronal processes. No immunoreactivity was observed in white matter, except for scattered ectopic ganglion cells. High NeuN labeling was observed in the animals of the SHAM groups 72 hours (Fig 10A) and 7 days (Fig 10D), since it is characterized by the marking of viable neurons. Non-treated animals subjected to neuropathic pain showed a significant decrease in the expression of marked neurons, more noticeable at 72 hours (Fig 10B) after injury. Treatment (CCI + MAR) demonstrated the ability to significantly increase the number of immunopositive cells (Fig 10M).

Regarding the expression of TACR1 in the spinal cord, it was observed that its expression was more accentuated in the injured group without treatment at 72 hours and 7 days, evidenced by the increase in immunopositive cells (Fig 10H and 10K), with a significant difference from SHAM group. Also, the treatment with maropitant was effective in reducing the area of expression of the receptor in the two evaluated moments (p<0.0001) (Fig 10I, 10L and 10N).

## Discussion

This study is the first to evaluate the therapeutic potential of maropitant through an experimental model of neuropathic pain induced by chronic constrictive injury of the sciatic nerve in rats. The results demonstrated a significant increase in MNT two hours after treatment administration. Furthermore, daily treatment was confirmed to improve damages caused by constrictive injury, leading to an increase in the expression of antioxidant enzymes in spinal cord. Additionally, it positively modulates oxidative stress, endoplasmic reticulum stress and immune mediators, characterizing the therapeutic potential of maropitant for the treatment of neuropathic pain.

The doses of maropitant used in this study were derived from previous studies conducted in mice. These studies indicated that a dose of 5 mg/kg was responsible for reducing lesions from ulcerative dermatitis [15]. Additionally, an 8 mg/kg dose was found to elicit an anti-inflammatory response in a model of acute induced pancreatitis [14], Furthermore, 30 mg/kg (IR) reduced the minimum alveolar concentration (MAC) associated with amitriptyline and minocycline, while 50 mg/kg (IR) caused secondary side effects [20].

The toxic effect of maropitant (50 mg/kg) observed in all animals in our study corroborates a pilot study, where such a dose was responsible for serious side effects, including the death of one animal [20].

When comparing the averages of mechanical sensitivity obtained prior to and seven days after CCI (Fig 2), a decrease in MNT was observed. The presence of mechanical hyperalgesia, quantified through the use of a digital analgesiometer, observed in this study was similar to those reported in other studies with the same experimental models [1, 21].

The development of NK1 antagonists for pain therapy has primarily focused on disrupting the neurotransmission of the SP from primary afferent nociceptors to central pain pathways in the dorsal horn [22]. Therefore, in the present study, a treatment cycle of 24 hours, for seven days was applied to monitor the clinical and analgesic effects of the medication throughout the treatment.

The intraperitoneal injection of maropitant led to increases in the nociceptive threshold during the initial days of treatment compared to the group that received saline solution. Studies have demonstrated the involvement of the NK1 receptor in a model of neuropathic pain induced by sciatic nerve constriction [23, 24]. Additionally, there is evidence that SP is responsible for the development of hypernociception in rats. It has been observed that blocking the receptors for SP (NK1) can revered hypernociception [25].

The increase in MNT observed in the animals from this study aligns with the results of other research. In those studies, treatments with NK1 receptor antagonist, such as i.e. L-733.060 or L-732.138, led to the attenuation of hyperalgesia. These findings indicate that SP and its NK1 receptor are involved both at the beginning and during the process of hyperalgesia persistence. This is further supported by evidence confirming the role of the SP in inducing pain [26].

Within 24 hours of drug injection, it was observed that MG30 showed better results than MG15, with a peak action between two to four hours after its application.

In addition to the improvement on MNT, treatment with maropitant reduced the gene expression of *HIF1α* and *NRF2* in injured rat spinal cords. These two biomarkers are associated with cellular hypoxia and oxidative DNA damage, respectively, and are transcription factor involved in the expression of antioxidant enzymes [27].

It is believed that the neuropathy model used in this study may have induced an increase in lipid peroxidation, followed by a compensatory decrease in the activities of SOD, CAT and GPx. Oxidative stress can alter both neurotransmission and neuronal function, damaging the integrity of the membrane due to increased membrane oxidation and presence of polyunsaturated fatty acids [28].

The treatment effectively regulated the SOD activity, reversed the stress inhibition effects on CAT, and resulted in a normal GPx activity. Consequently, the protective effect of the medication may be due attributed to its antioxidant action. Taken together, these findings suggests that maropitant has a protective effect against hypoxia and oxidative stress in the spinal cord caused by sciatic nerve injury. Furthermore, maropitant, which has not been evaluated *in vivo* so far for peripheral nerve injury, exhibits cytoprotective effects against oxidative stress.

These results align with studies demonstrating the disruption of proper functioning of ER induced by neuropathic pain, thereby causing ER stress, which severely impairs the normal protein folding process. Cells respond to ER stress by increasing the expression of molecular chaperones such as GRP 78, mediated by ER stress receptors (PERK and CHOP), initiating a defensive process called the Unfold Protein Response (UPR) [29].

In addition to assessing the protective potential of maropitant against oxidative stress in the spinal cord caused by peripheral nerve injury, we also analyzed its protective effects against endoplasmic reticulum stress, another biological process involved in cell damage that can be caused by oxidative stress [30].

Until now, endoplasmic reticulum stress had not been studied in *in vivo* models evaluating maropitant effects. It was observed that the treatment reduced neuronal gene expression of *GRP78*, *CHOP* and *PERK* in the dorsal horn of the spinal cord. Based on these results, it may be proposed that the downregulation of junction proteins can be attributed to protective effects of maropitant in the spinal cord of rats with neuropathic pain caused by constrictive injury.

Considering that the activation of oxidative stress and endoplasmic reticulum stress can alter the expression of immunological mediators [31], the expression of IL_10_, TNFα and IBA-1 was also evaluated.

The induction of peripheral neuropathic pain in animals had the ability to modulate central levels of cytokines. This aligns with a previous study where intrathecal injection of oxaliplatin was applied. In that study, inflammation and oxidative stress were responsible for the pathophysiological disturbance of neuropathic pain [32].

The results from this study showed an increased expression of IL_10_ and TNF-α in injured animals without treatment. This aligns with other studies that have demonstrated increased expression of cytokines in various neuropathic pain models, such as chronic sciatic nerve damage [33].

The treatment performed in this study effectively normalized the levels of IBA-1 levels, thus preventing secondary events that would be triggered under conditions of exacerbated microglia activation. The release of inflammatory cytokines, neurotrophic factors, prostaglandins, and ROS, all of which excite nociceptive neurons in the spinal cord, contributes to the persistence of chronic pain [34, 35].

The known mechanisms of neuropathic pain are the main target of drug studies focused on pain relief. Currently, there are still limited studies successfully describing the relationships between the NK1 and its receptor in this pathological process. Here, it was demonstrated that the animals with lesions had higher IBA-1 marking on the spinal cords compared to control group. As is known, microglial cells are present under normal physiological conditions; nevertheless, their activation may be exacerbated during pathological processes [36].

Maropitant was responsible for decreasing the microglia activation, suggesting that its activation is related to the substance P and its receptor. Previous work has found that NK1 glial receptors may play a key role in pain expression mediated through the primary afferent nociceptive pathways in the spinal cord [37].

Additionally, it was found that the microglia act not only through promoting antigen presenting cells but also on effector cells, which may cause damage to other cells in the central nervous system directly *in vitro* and *in vivo* [38]. Consequently, the inhibition of microglial activation during treatment might be considered a therapeutic option for neurodegenerative diseases.

Regarding the expression of NeuN in the spinal cord, it was observed that the CCI model induced a decrease in its production. That is justified by the fact that TNF-α binds to the TNF-α receptor in Schwann cells, causing the induction of apoptosis [39]. High levels of TNF-α causes intensification of neuronal death in rats’ spinal cord [40, 41].

Since TNF-α is the key cytokine capable of stimulating the extensive release of microglial glutamate in an autocrine manner, inducing neurotoxicity [42], increased levels of TNF-α are correlated with the increase of neuronal death, an outcome demonstrated in this study. Treatment with NK1 antagonist prevented neuronal death, as evidenced by the increased expression of marked neurons in the animals spinal cord. Notably, inflammatory modulation (significant positive correlation) with the use of the NK1 antagonist was observed, as it was able to protect against neurogenic inflammation induced by peripheral nerve damage.

## Conclusions

Treatment with maropitant at a dose of 30 mg/kg/24q protects against hypoxia, oxidative stress and endoplasmic reticulum stress caused by neuropathic pain in neurons of the dorsal horn in spinal cord. Therefore, this reduction in central/peripheral sensitization leads to a decrease in allodynia and hyperalgesia. The interruption of SP binding to the NK1 receptor, mediated by maropitant, provides a new perspective on that intracellular pathway, representing a potential target for further research in the search for new treatment strategies for patients suffering from neuropathic pain.

## Supporting information

S1 File (PZF)

S2 File (XLSX)
